# High expression levels and the C3435T SNP of the *ABCB1* gene are associated with lower survival in adult patients with acute myeloblastic leukemia in Mexico City

**DOI:** 10.1186/s12920-021-01101-y

**Published:** 2021-10-26

**Authors:** Irma Olarte Carrillo, Anel Irais García Laguna, Adrián De la Cruz Rosas, Christian Omar Ramos Peñafiel, Juan Collazo Jaloma, Adolfo Martínez Tovar

**Affiliations:** 1 Laboratorio de Biología Molecular, Servicio de Hematología, Hospital General de México, “Dr. Eduardo Liceaga”, Ciudad de México, Mexico; 2 Servicio de Hematología, Hospital General de México, “Dr. Eduardo Liceaga”, Ciudad de Mexico, México

**Keywords:** *ABCB1*: ATP binding cassette subfamily B member 1, qRT-PCR: quantitative real-time polymerase chain reaction, AML: acute myeloid leukemia

## Abstract

**Background:**

Acute myeloid leukemia (AML) is a heterogeneous hematologic malignancy characterized by different genetic alterations that cause changes in the normal mechanisms of differentiation, which are associated with chemoresistance. The *ABCB1* gene is part of a family of ATP-binding cassette (*ABC*) transporter genes involved in the progression of various types of cancer. The following work aimed to evaluate the expression levels of the *ABCB1* gene and the C3435T SNP with the response to first-line treatment and survival in patients with AML.

**Methods:**

In total 135 samples were taken to isolate total RNA and DNA at the beginning of the treatment. Expression analysis by RT-qPCR and SNP C3435T assessment method were performed for real-time Polymerase chain reaction (qPCR).

**Results:**

The expression levels impact on the survival of patients with AML compared to low or absent levels; the CC genotype was found in 22.9%, the CT genotype was found in 47.4%, and the TT genotype was found in 29.6%, the presence of the C3435T SNP, the TT genotype also impacts with a lower survival compared to CT and CC genotypes. In addition, it was shown that the dominant model significantly impacts survival.

**Conclusion:**

In conclusion, we have found that the overexpression of the *ABCB1* gene, as well as the presence of the TT genotype of the C3435T SNP, contributes to a worse prognosis in AML.

## Background

Acute myeloid leukemia (AML) is the most frequent in adults, it is a myeloproliferative disorder with a high risk of relapse and a high mortality rate [1, 2]. In adult Mexican patients with AML, the median age is 32 years, less than other international series [3]. With the best treatment regimens (BMT, immunotherapy, and targeted molecular therapy), most patients with AML can achieve complete remission (CR) [4, 5]; however, 5-year survival in our country is only 25% of patients with AML [6, 7]. The molecular mechanisms that cause therapy failure leading to an unfavorable prognosis in AML are still not fully understood and are one of the most difficult obstacles in therapy [8].One of the common mechanisms for treatment failure is overexpression of drug resistance genes (*MDR-1/ABCB1*: ATP-binding cassette transporters) [9, 10]. The gene is located on chromosome 7q21.1 and is made up of 28 introns and 28 exons. *ABCB1* mRNA is 4.7 kb, they encode a 170 kDa membrane transporter called P-glycoprotein (P-gp) [11, 12]. This gene has been studied extensively in search of polymorphisms; to date, around of 50 SNPs have been identified for *ABCB1* [13]. The most studied polymorphisms are: C1236T (rs1128503), G2677T / A (rs2032582), and C3435T (rs1045642) [14, 15]. C3435T SNP is synonymous C to T polymorphism at nucleotide position 3435 in exon 26 (3435 C > T) [16]. This transition does not change the amino acid encoded with Ile at position 114,522; the TT variant has been associated with the decrease in the expression and with the stability of the protein as the transport of the drugs [17]. It has been described that the effect of the polymorphism can affect the post-transcriptional processing of mRNA by interfering with the intron removal process, as well as by affecting the process of alternative transcription splicing [18]. It has been reported that C3435T SNP and P-gp expression levels in AML patients could be associated with prognosis and the survival and relapse in AML patients [19]. Based on the above, we evaluated the frequency of the C3435T SNP, as well as the expression levels of the *ABCB1* gene, associated with treatment response and survival in patients with AML.

## Methods

### Study population

In total, 135 patients with AML were analyzed at diagnosis, trials were taken into preservative-free heparin or into EDTA tubes following informed consent as approved by local and national ethics committees and sent laboratories in the Hospital General de Mexico, with a complete clinical record over a 4-year period (2016–2020). The sample size calculation was carried out through G-Power to obtain 80% of the effect size; we agree that the number of patients is low, but with the results obtained, the study can be expanded to include a more significant number of affected individuals.

The mean age was 47 years (15–92 years). Regarding gender, 48% were female (n = 65) and the rest male (n 70, 51.9%). The mean hemoglobin was 10.6 g/dl, (2.6–91 g/dl) with a mean Leukocyte of 154 × 109/ L (0.2–456 × 109/L) and 46 × 109/L for platelets (3–241 × 109/L). Regarding the main genetic alterations identified, 50.4% had a normal karyotype. The mean overall survival of the patients was 193 days (164–222), (Table 1). This study was approved by Ethics Committee of Hospital General de Mexico “Dr. Eduardo Liceaga”. Written informed consents to participate were obtained from all the participants in this study (written informed consent to participate of individuals younger than the age of 16 were obtained from their parents or legal guardians).

**Table 1:** Clinical characteristics of the population analyzed n = 135

*Clinical features*	
Age	
Mean ± SD (range)	47 (15–92)
Median	48
Sex	
F	65 (48.1)
M	70 (51.9)
*Laboratory data*	
PB Blast count	
Mean ± SD (range)	58 ± 28 (0–99)
Median	60
Mean WBC count, 10^9^/L (range)	154 (0.2–456)
Mean hemoglobin level, g/L (range)	10.61 (2.60–91)
Mean platelet count, 10^9^/L (range)	46 (3–241)
Mean DHL (range)	635 (89–3921)
*Biologic characteristics*	
Immunophenotype (%)	
M1	4 (3.0)
M2	42 (31.1)
M4	81 (60.0)
M5	2 (1.5)
M6	4 (3.0)
M7	2 (1.5)
Cytogenetics	
Unsuccessful karyotype	63(46.7)
Normal karyotype	68(50.4)
Abnormal karyotype	4 (3.0)
Treatment scheme	
3 + 7	108 (80)
Mini 3 + 7	16 (11.9)
2 + 5	3 (2.2)
ARA C SC	8 (5.9)
Response	
Response complete	55 (40.7)
Remission partial	16 (11.9)
Refractory disease	28 (20.7)
Death in aplasia	30 (22.2)
Death by undetermined cause	6 (4.40)

### Type of treatment

The treatment was based on the 7 + 3 scheme, the intensity of the treatment was mainly based on the age and functional status of the patients. The 7 + 3 normal intensity scheme (cytarabine 100 m /m2 for 7 days plus daunorubicin 60 mg/m2 for 3 days) was started in 108 patients (n = 80%), a total of 16 patients received reduced 7 + 3 doses (11.9%) and 7 patients received subcutaneous cytarabine (6.9%).

### Response to treatment

The response to treatment was assessed considering the recovery of the hematic biometry parameters, the treatment was checked at day 28 based on bone marrow uptake. Complete Remission was defined as the patient having less than 5% of blasts at the end of induction therapy, Refractory patients remained leukemic, and Therapeutic Failure was defined as the patient dying during therapy. Patients who had complete remission and who presented an increase in the number of blasts (> 5%) at any time were considered to be in Relapse. The consolidation phase consisted of the administration of sequential blocks of chemotherapy, including administration of high doses of methotrexate. At the end of the study, the patients started the maintenance phase by administering weekly mercaptopurine and methotrexate for a duration of 2 years. In case of relapse to bone marrow, the patients received rescue therapy [20]. The evaluation of the minimal residual disease was evaluated at the hematological level. A total of 55 patients (40.7%) had Complete Remission criteria, while 16 patients (11.9%) were considered as Partial Response. Regarding refractory leukemia, 20.7% (n = 28) showed resistance to the first treatment scheme, while 26.6% (n = 36) died during the remission induction stage, 30 cases due to aplasia and 6 cases by indeterminate death.

### Detection of SNP C3435T

#### Determination of single nucleotide polymorphisms (SNPs) was analyzed using real-time

Polymerase chain reaction (qPCR) by 48-well plate Step One Real Time PCR system (Applied Biosystems, Carlsbad, CA, USA) with TaqMan probes by Applied Biosystems Step One™. The SNP probes 3435C > T (rs1045642), of the *ABCB1* gene. Master mixes were prepared as recommended by the manufacturer. 10 ng of DNA samples were added to each well and the reaction was carried out in a Step one Detection System (Life Technologies). The PCR conditions comprised an initial denaturation step of 10 min at 95 °C, followed by 5 cycles of 15 s denaturation at 95 °C, and one minute extension at 58 °C. This was followed by 40 cycles of 15 s denaturation at 95 °C and one minute extension at 60 °C. Real time data were collected during the last 40 cycles of amplification. They were evaluated for the dominant genetic model (CT + TT vs. CC) and the recessive genetic model (TT vs. CC + CT) [22]. The cell line k562, which presents the allele TT and cell line Molt4 with CT allele and the Jurkat cell line for CC of SNP C3435, were used as positive control.

### Determination of the *ABCB1* gene

All samples were collected from the bone marrow, and the mononuclear density (1.077 g/L). The mononuclear cell phase was separated and suspended in PBS medium and stored at − 70 °C. RNA Isolation was performed using TRIzol® (Invitrogen/Life Technologies). The RNA was stored at − 80 °C until needed. For cDNA synthesis, 2 μg of RNA final volume of 20μL was combined with 200U of the MMLV RT enzyme (Invitrogen, Carlsbad, CA, USA) [21].

### Real-time polymerase chain reaction

The mRNA expression levels of the *ABCB1* (Hs01069047) and glyceraldehyde 3-phosphate dehydrogenase (GAPDH; Hs00985689) genes were measured using TaqMan® gene expression assays (Applied Biosystems. Foster City, CA, USA). The GAPDH gene was used as an endogenous control, and each sample was analyzed in triplicate. The relative expression levels were calculated using the2 − ΔΔCt method. The high and low expression cutoff points were determined based on the mean values observed in 100 healthy donors. The RT-PCR conditions comprised an initial denaturation step of 10 min at 95 °C, followed by 60 °C 30″, 95 °C 10′; 30 cycling 95 °C 15′, 60 °C 1′ [22].

### Statistical analysis

The multivariate analysis was performed based on clinical parameters, in the presence of C3435T SNP. A Kaplan Meier and Long Rank analysis was performed to assess survival in relation to each SNP, differences with p < 0.05 were considered significant by means of the SPSS Software, Version 20.0 (Statistical Package for Social Sciences, SPSS Inc, Chicago ILL, USA).

## Results

The relative expression levels of the *ABCB*1 gene were analyzed in 135 patients and 99 healthy donors. The results showed high expression, with a sixfold difference in *ABCB1* (p = 0.001) from the group of healthy donors. The frequencies of patients with AML with high expression levels of the *ABCB*1 gene were 34.8% (47/135) Mean ± SD (range) 0.97 ± 15(0.65–1.9) median (0.94) and 37.7% (51/135) Mean ± SD (range) 0.48 ± 15(0.23–0.6) medians (0.45) presented low levels. No association was found between the clinical parameters and the ABCB1 gene expression levels and SNP C3435T (Tables 2, 3).

**Table 2:** Significance of the expression gene *ABCB1*

	Negative	Low	High	p value
*Age*				
< 60	26	38	29	
> 60	11	13	18	.383
*Gener*				
Femenine	17	22	26	
Male	20	29	21	.460
*Blast count*				
< 40	15	15	15	
40–60	4	12	8	
< 60	18	24	24	.575
*WBC count, 10*^*9*^*/L (range)*				
< 35	24	33	27	
35–100	8	13	10	
> 100	5	5	10	.605
*Immunophenotype*				
M1	1	1	2	
M2	13	15	14	
M4	20	32	29	
M5	1	1	0	
M6	2	0	2	
M7	0	2	0	.631
*Cytogenetics*				
Unsuccessful karyotype	17	26	20	
Normal karyotype	20	23	25	
Abnormal karyotype	0	2	2	.671
*Response*				
Response complete	13	28	14	
Remission partial	5	4	7	
Refractory disease	8	8	12	
Death in aplasia	9	8	13	
Death by undetermined cause	2	3	1	.338

Pearson’s Chi Square Test

**Table 3:** Significance of the SNP *ABCB1* C3435T gene

	CC	CT	TT	p Value
*Age*				
≤ 60	21	44	28	
> 60	10	20	12	.979
*Gener*				
Female	13	28	24	
Male	18	36	16	.199
*Blast count*				
< 40	10	20	15	
40–60	6	15	3	
> 60	15	29	22	.361
*WBC count, 10*^*9*^*/L (range)*				
< 35	14	40	30	
35–100	12	14	5	
> 100	5	10	5	.092
*Immunophenotype*				
M1	0	2	2	
M2	10	18	14	
M4	20	38	23	
M5	1	1	0	
M6	0	3	1	
M7	0	2	0	.719
*Cytogenetics*				
Unsuccessful karyotype	18	26	19	
Normal karyotype	12	37	19	
Abnormal karyotype	1	1	2	.411
*Response*				
Response complete	12	26	17	
Remission partial	6	5	5	
Refractory disease	3	15	10	
Death in aplasia	7	15	8	
Death by undetermined cause	3	3	0	.372

Pearson’s Chi Square Test

**Fig. 1:**
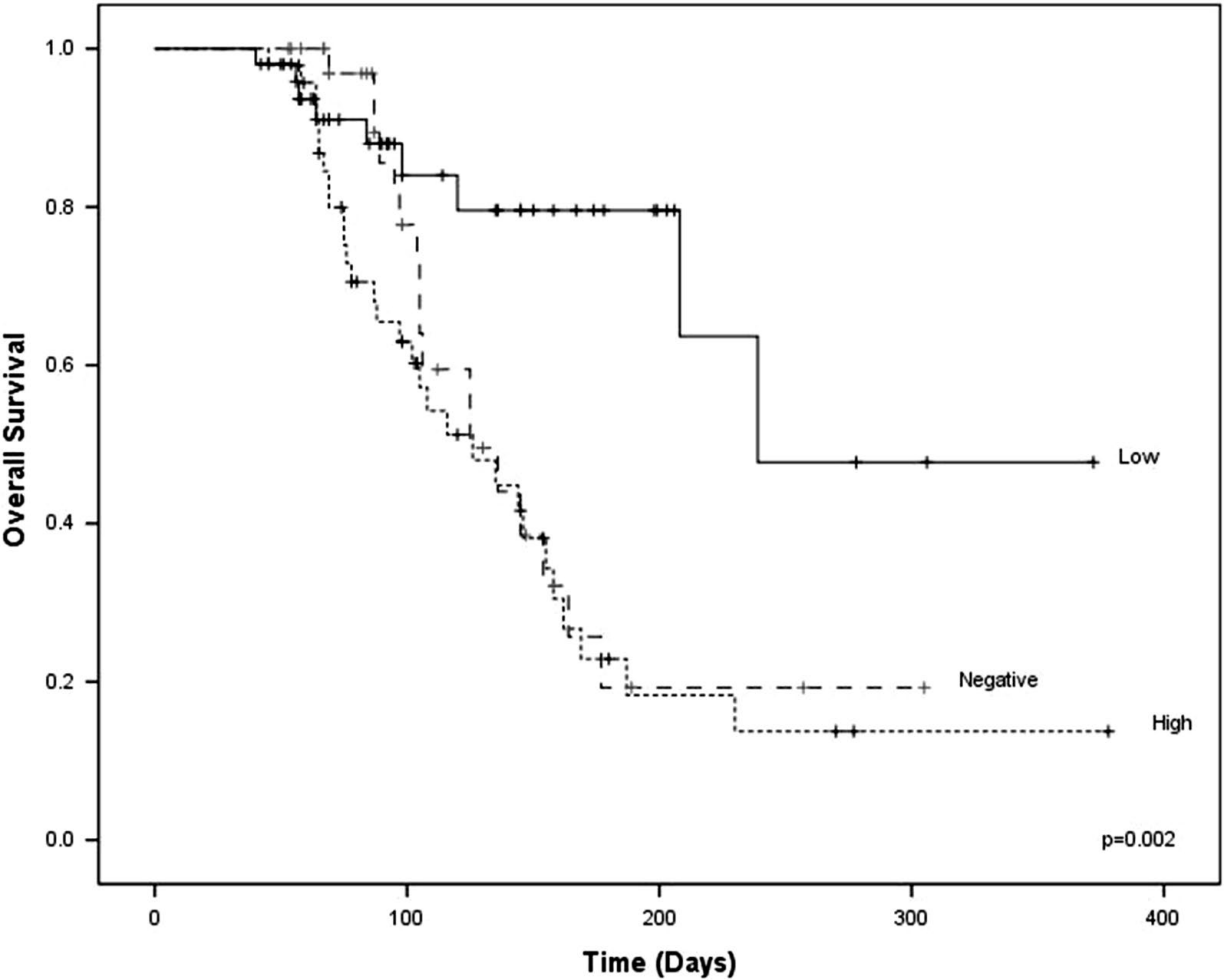
Significant decrease in OS in patients with high expression levels of the *ABCB1* gene log rank p = 0.002

We demonstrated that at an overall survival (OS) of 400 days, low expression had OS values of 82% (42/51) with a mean of 265.4 days (204.9–325.9), while high-expression and negative patients had a lower survival with 36.2% (17/47) mean of 152.9 days (117.8–187.98), and 54% (20/37), with a mean of 157.2 (122.9–191.4) respectively. The results indicated a significant decrease in OS in patients with high expression levels of the *ABCB1* gene log rank p = 0.002, (Fig. 1).

When analyzing the most frequent polymorphism of the C3435T SNP of the *ABCB1* gene in patients with AML, the CC genotype was found in 22.9%, the CT genotype was found in 47.4%, and the TT genotype was found in 29.6%.

**Fig. 2:**
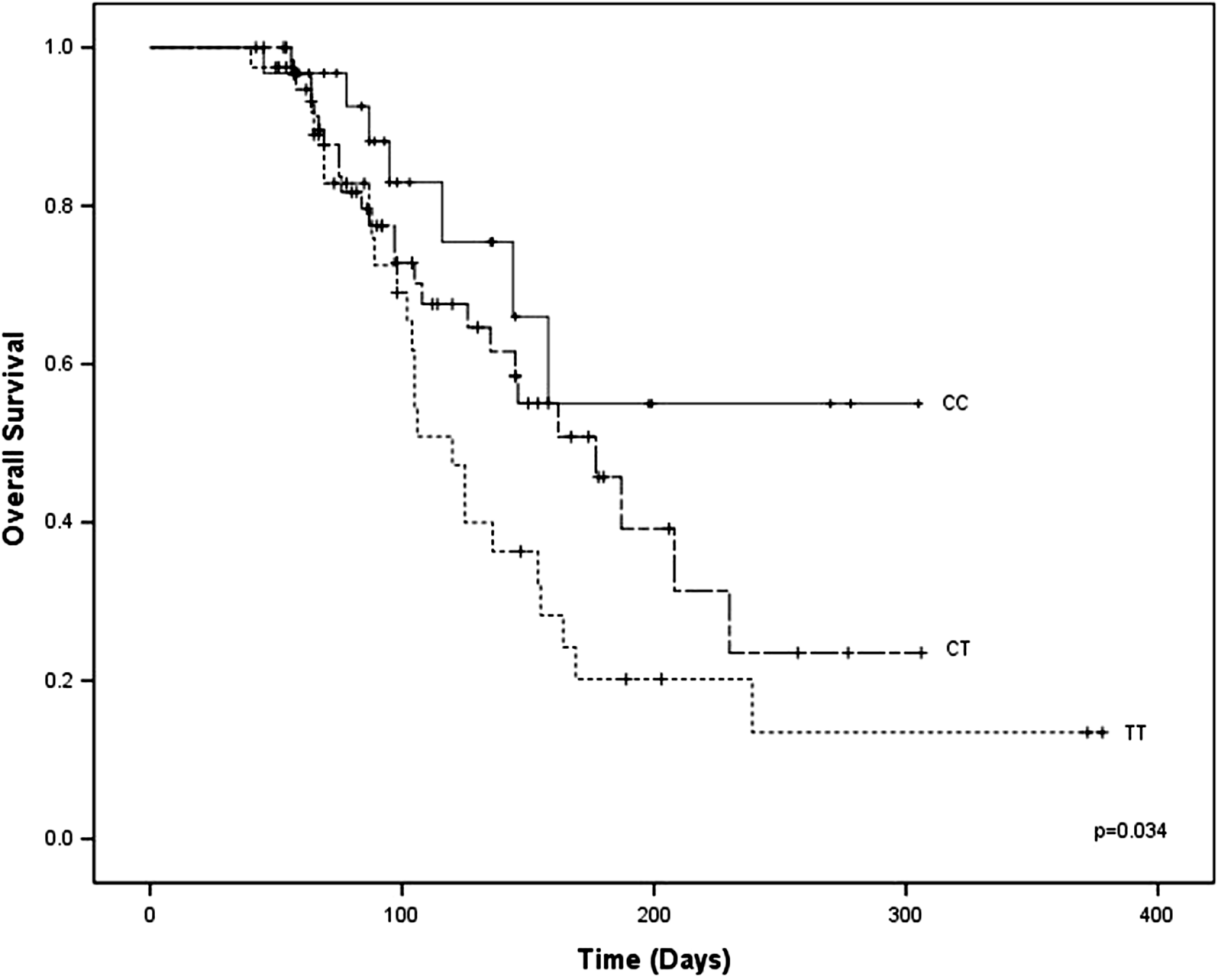
Significant decrease in OS in patients the presence of C3435T SNP

When analyzing OS with the presence of C3435T SNP, the mean survival time was shorter in patients with a TT genotype of 120 days (102.4–137.5) compared to the CC genotype of 220 days (170.8–271), and CT of 177 (131.53–222.56) log-rank (p = 0.034) (Fig. 2).

**Fig. 3:**
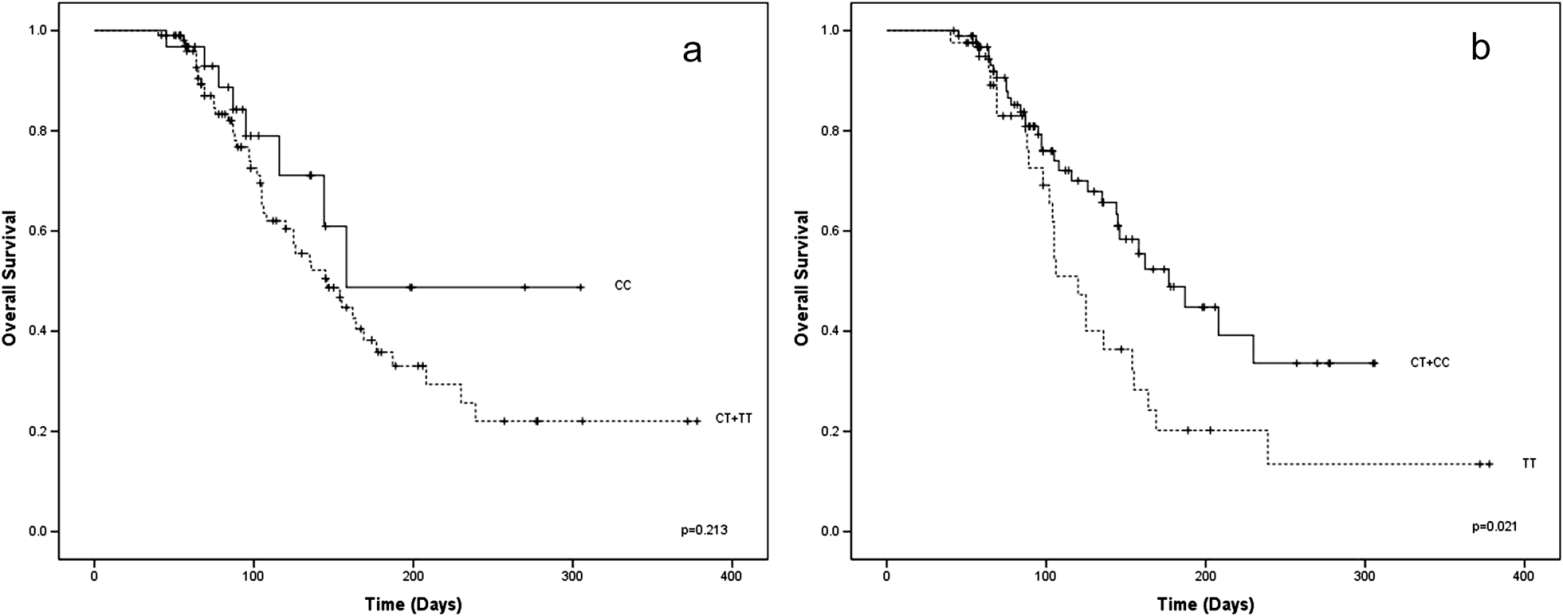
Significant in OS in patients with the presence of the recessive and dominant models of SNP C3435T

**Fig. 4:**
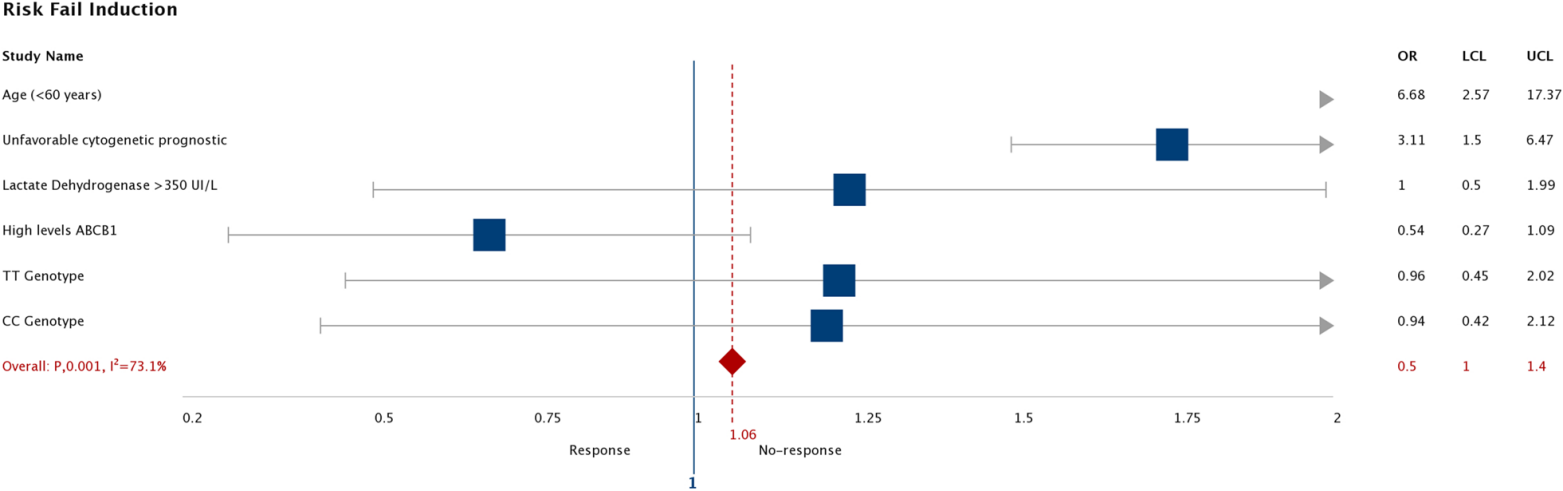
Evaluating the risk of treatment failure. *OR* Odds Ratio, *UCL* Upper Confidence Limit (límite superior de confianza), *LCL* Lower Confidence Limit (límite inferior de confianza)

**Fig. 5:**
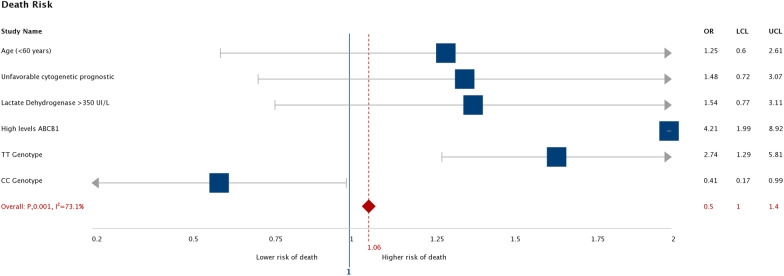
Evaluating the risk of Death. *OR* Odds Ratio. *UCL* Upper Confidence Limit (límite superior de confianza), *LCL* Lower Confidence Limit (límite inferior de confianza)

In the recessive and dominant models of SNP C3435T, the recessive TT was found to have an OS of 153 days (115–191) compared to CT + CC of 191 days (164–218), log rank (p = 0.021). In the case of the dominant model, the TC + TT combination was 183 days (153.2–214.2) versus CC 207 days (155–260), log-rank (p = 0.213), Fig. 3. When evaluating the risk of treatment failure, only age older than 60 and unfavorable cytogenetic prognosis were significant (OR 6.68; 95% CI: 2.56–17.36) and (OR 3.11; 95% CI: 1.49–6.46) respectively. Regarding the risk of death, the high levels of expression of the *ABCB1* gene (OR 4.2; 95% CI: 1.98–8.91) and the TT genotype (OR 2.7; 95% CI: 1.28–5.81) presented statistical significance (Figs. 4 and 5).

## Discussion

Resistance to treatment remains a major obstacle in AML, especially in young patients who represent a population with curative potential and long-term remission after intensive treatment; however, 30% of these patients with AML survive to 5 years with the best treatment schemes [23]. One of the mechanisms of resistance to treatment reported is the overexpression of drug resistance genes, causing unfavorable results and death [24, 25]. Overexpression of the gene and function of the glycoprotein (P-gp) can be altered by the presence of polymorphisms. The SNP 3435C > T (rs1045642) in exon 26, alters gene expression, protein activity, and substrate specificity [24]. Therefore, in this work, we examined whether the expression levels of the *ABCB1* gene is affected due to the presence of C3435T SNP and its relationship with the prognosis and overall survival in adult AML patients [26, 27].

It was found that only 36% of the population had high levels of expression relative, 0.94 medians, range (0.65–1.9) of the *ABCB1* gene, in patients with AML; several previous AML studies have shown that *ABCB1 high* level are expressed from 35 to 70% in adult cases [28, 29]. Regarding the high levels of expression and OS, a significant association was found; this is similar to that described by Boyer et al. 2019, where they showed that in a cohort of 161 patients with AML, a low level expression, 0.45 medians, range (0.23–0.6) of the *ABCB1* gene a had higher OS [30]. In this hematological neoplasm, it has been reported that the rate of complete remission and drug resistance are related to the function and expression of *ABCB1* [25]. It has been described that the expression and functional activity of the *ABCB1* gene increases with the age of the patient, from the 17% in patients under 35 years of age up to 39% in patients 50 years of age or older [13]. In our population, the average age was 32 years, with a higher frequency of expression than that reported internationally, and this could have repercussions with the observed OS.

The frequency of the C3435T SNP of the *ABCB1* gene population was 77% for the CC genotype, while the TT genotype was 40% in our study. These results are similar to those reported by other authors in AML and ALL [31, 32]. Regarding the association with clinical parameters, it has been reported that there is no association in the recessive and dominant models, which is in agreement with our findings. However, in solid cancer, the association with clinical parameters is different from that reported by Tazzite A, et al. where they found a significant correlation between the *ABCB1* C3435T polymorphism and the clinical grades of breast cancer [33].

The possibility that other polymorphisms combine with the C3435T SNP to induce an effect on *ABCB1* levels could be one of the reasons for this controversy. Several studies have shown a decrease with the expression of the *ABCB1* gene and the presence of CT in patients with AML^.^ [34, 35]. On the contrary, there are other studies that relate the TT or CC polymorphism with a lower expression of the *ABCB1* gene [36]. Other authors mention that the expression of *ABCB1* in patients with AML is independent of the presence of the C3435T genotype [31]. Our results showed that there is no association between the expression levels of the *ABCB1* gene and the presence of the C3435T SNP, so it seems to be an independent mechanism. The increase in expression levels may be due to the fact that chemotherapeutic agents by themselves induce an increase in the expression of transporter genes or an increase in transcription activity, and not necessarily because of the presence of some of the TT genotypes or CT of C34345T SNP. Another explanation is that the C3435T SNP is out of balance with other non-coding polymorphisms, such as G2677T and C1236T, which are part of a common haplotype [35].

This SNP has been reported to play an important role in the efflux membrane pump of the P-glycoprotein, protecting cells and organs against xenobiotic agents and environmental carcinogens [37]. Therefore, the presence of the TT polymorphism affects transportation and the different types of treatments used in this neoplasm. In this study, we found that the TT genotype of C3435T SNP was associated with a lower OS. Thomas I. et., 2002 reported in 405 patients with AML that the CC genotype was associated with a lower OS [38]; however, other studies, such as that of Holt B. et.al. and that of Jamroziak K. et al., reported that the TT genotype of C3435T SNP does not significantly affect the clinical prognosis of patients with AML [39, 40]. These differences in the reports may be due to nutritional factors, sample size, methodology used and the type of ethnicity.

When evaluating the OR in our patients, a 4.2 times higher risk of death was demonstrated when *ABCB1 l*evels are high and 2.7 times when the TT genotype is found. Various studies have shown that not all SNPs are considered silent; they can cause changes in the expression, conformation, or function of proteins and are increasingly implicated in the risk of human diseases [41].

Allele frequencies of the *ABCB1 g*ene, C3435T polymorphism have been evaluated around the world, and significant inter-population differences have been detected (Leal-Ugarte et al. 2008) [42, 43]. According to the literature, the frequency of the T allele in Caucasian and Asian populations is about 50% for each [44].

## Conclusion

We have proved that the overexpression of the *ABCB1* gene, as well as the presence of the TT genotype of the C3435T SNP, adds to the outcome markers already known as FLT3, IDH, DNTM3, contributing to a worse prognosis. Therefore, detecting the levels and C3435T SNP in our population of patients with AML at diagnosis can help predict prognosis.

## Data Availability

The datasets used and/or analysed during the current study available from the corresponding author on reasonable request.

## Abbreviations

AML: Acute myeloid leukemia
*ABCB1*: ATP-binding cassette B1
qPCR: Real-time Polymerase chain reaction
BMT: Targeted molecular therapy
CR: Achieve complete remission
P-gp: P-glycoprotein
SNP: Single nucleotide polymorphisms
OS: Overall survival
